# Supplementary material for: A Draft Genome of the Ginger Species Alpinia nigra and New Insights into the Genetic Basis of Flexistyly
Source: Genes (Basel). 2021 Aug 24;12(9):1297. doi: 10.3390/genes12091297 (PMC8468202; doi:10.3390/genes12091297)
Supplement: Supplementary file 1 [file genes-12-01297-s001.zip › Supplementary_information_flexistyly.pdf]

Table S1: Collection details of the anaflexistylous and cataflexistylous leaf tissue samples of *Alpinia nigra* from Pakke Tiger Reserve, Arunachal Pradesh, India collected on 09/07/2018.

Tissue no.	Collection no.	Latitude	Longitude	Elevation	Morph type
1	SR015_18	N 26°56'59.9"	E 92°59'18.9"	136m	Ana
2	SR015_18	N 26°57'00.2"	E 92°59'18.7"	156m	Ana
3	SR015_18	N 26°57'00.1"	E 92°59'19.4"	133m	Ana
4	SR015_18	N 26°57'01.0"	E 92°59'19.4"	134m	Ana
5	SR015_18	N 26°57'01.9"	E 92°59'19.5"	134m	Ana
6	SR015_18	N 26°57'02.1"	E 92°59'19.6"	134m	Ana
7	SR015_18	N 26°57'02.0"	E 92°59'19.7"	134m	Ana
8	SR015_18	N 26°57'02.4"	E 92°59'20.1"	134m	Ana
9	SR015_18	N 26°57'02.6"	E 92°59'20.0"	134m	Ana
10	SR015_18	N 26°57'03.0"	E 92°59'20.6"	134m	Ana
11	SR015_18	N 26°57'02.8"	E 92°59'20.9"	133m	Ana
12	SR015_18	N 26°57'03.0"	E 92°59'21.1"	133m	Ana
13	SR015_18	N 26°57'03.3"	E 92°59'21.2"	133m	Ana
14	SR015_18	N 26°57'03.5"	E 92°59'21.2"	133m	Ana
15	SR015_18	N 26°57'03.7"	E 92°59'21.2"	133m	Ana
16	SR015_18	N 26°57'03.8"	E 92°59'21.4"	133m	Ana
17	SR015_18	N 26°57'03.9"	E 92°59'21.6"	133m	Ana
18	SR015_18	N 26°57'04.0"	E 92°59'21.4"	133m	Ana

19	SR015_18	N 26°57'04.1"	E 92°59'20.9"	132m	Ana
20	SR015_18	N 26°57'04.5"	E 92°59'21.6"	133m	Ana
21	SR015_18	N 26°57'04.6"	E 92°59'21.4"	133m	Ana
22	SR015_18	N 26°57'05.1"	E 92°59'21.5"	133m	Ana
23	SR015_18	N 26°57'05.2"	E 92°59'21.4"	132m	Ana
24	SR015_18	N 26°57'04.9"	E 92°59'22.3"	132m	Ana
25	SR015_18	N 26°57'04.9"	E 92°59'22.6"	132m	Ana
26	SR015_18	N 26°57'05.5"	E 92°59'22.9"	133m	Ana
27	SR015_18	N 26°57'05.5"	E 92°59'23.4"	133m	Ana
28	SR015_18	N 26°57'05.7"	E 92°59'23.6"	133m	Ana
29	SR015_18	N 26°57'05.8"	E 92°59'24"	133m	Ana
30	SR015_18	N 26°57'05.8"	E 92°59'25.5"	133m	Ana
31	SR015_18	N 26°57'07.3"	E 92°59'28.7"	130m	Ana
32	SR015_18	N 26°57'07.3"	E 92°59'29"	128m	Ana
33	SR015_18	N 26°57'08.2"	E 92°59'29.3"	128m	Ana
34	SR015_18	N 26°57'08.3"	E 92°59'30.2"	128m	Ana
35	SR015_18	N 26°57'08.2"	E 92°59'30.5"	129m	Ana
36	SR015_18	N 26°57'16.8"	E 92°59'32.7"	129m	Ana
37	SR015_18	N 26°57'12.1"	E 92°59'33.3"	121m	Ana
38	SR015_18	N 26°57'11.7"	E 92°59'33.4"	122m	Ana

39	SR015_18	N 26°57'10.1"	E 92°59'33.1"	123m	Ana
40	SR015_18	N 26°56'24.1"	E 92°59'00.8"	146m	Ana
41	SR015_18	N 26°56'28.9"	E 92°59'03.3"	121m	Ana
42	SR015_18	N 26°56'29.5"	E 92°59'04.9"	120m	Ana
43	SR015_18	N 26°56'31.1"	E 92°59'04.7"	121m	Ana
44	SR015_18	N 26°56'31.6"	E 92°59'05.8"	122m	Ana
45	SR015_18	N 26°56'32"	E 92°59'06.6"	122m	Ana
46	SR015_18	N 26°56'38.5"	E 92°59'08.9"	122m	Ana
47	SR015_18	N 26°56'38.8"	E 92°59'10.1"	122m	Ana
48	SR015_18	N 26°56'39"	E 92°59'10.8"	122m	Ana
49	SR015_18	N 26°56'39.6"	E 92°59'11.6"	122m	Ana
50	SR015_18	N 26°56'51.9"	E 92°59'13.2"	126m	Ana
51	SR015_18	N 26°57'00.0"	E 92°59'19"	128m	Ana
1	SR015_18	N 26°57'00.1"	E 92°59'18.7"	207m	Cata
2	SR015_18	N 26°57'00.2"	E 92°59'19"	138m	Cata
3	SR015_18	N 26°57'00.2"	E 92°59'19"	138m	Cata
4	SR015_18	N 26°57'00.3"	E 92°59'19.3"	134m	Cata
5	SR015_18	N 26°57'01.1"	E 92°59'19.2"	135m	Cata
6	SR015_18	N 26°57'01.5"	E 92°59'19.7"	135m	Cata
7	SR015_18	N 26°57'02.0"	E 92°59'19.7"	135m	Cata

8	SR015_18	N 26°57'01.9"	E 92°59'19.8"	134m	Cata
9	SR015_18	N 26°57'02.4"	E 92°59'20.2"	134m	Cata
10	SR015_18	N 26°57'02.5"	E 92°59'20.0"	134m	Cata
11	SR015_18	N 26°57'03.0"	E 92°59'20.4"	134m	Cata
12	SR015_18	N 26°57'03.0"	E 92°59'20.7"	133m	Cata
13	SR015_18	N 26°57'03.1"	E 92°59'21.1"	133m	Cata
14	SR015_18	N 26°57'03.5"	E 92°59'21.2"	133m	Cata
15	SR015_18	N 26°57'03.5"	E 92°59'21.4"	133m	Cata
16	SR015_18	N 26°57'03.7"	E 92°59'21.2"	133m	Cata
17	SR015_18	N 26°57'03.7"	E 92°59'21.2"	133m	Cata
18	SR015_18	N 26°57'03.9"	E 92°59'21.6"	133m	Cata
19	SR015_18	N 26°57'04.1"	E 92°59'21.3"	133m	Cata
20	SR015_18	N 26°57'04.5"	E 92°59'21.5"	133m	Cata
21	SR015_18	N 26°57'04.9"	E 92°59'21.6"	132m	Cata
22	SR015_18	N 26°57'05.1"	E 92°59'21.4"	132m	Cata
23	SR015_18	N 26°57'05.2"	E 92°59'21.4"	133m	Cata
24	SR015_18	N 26°57'05.4"	E 92°59'21.5"	133m	Cata
25	SR015_18	N 26°57'05.5"	E 92°59'21.5"	133m	Cata
26	SR015_18	N 26°57'05.6"	E 92°59'21.8"	132m	Cata
27	SR015_18	N 26°57'05.2"	E 92°59'22.2"	132m	Cata

28	SR015_18	N 26°57'05.0"	E 92°59'22.9"	132m	Cata
29	SR015_18	N 26°57'05.2"	E 92°59'23"	133m	Cata
30	SR015_18	N 26°57'05.6"	E 92°59'23"	133m	Cata
31	SR015_18	N 26°57'05.5"	E 92°59'23.4"	133m	Cata
32	SR015_18	N 26°57'05.8"	E 92°59'23.4"	133m	Cata
33	SR015_18	N 26°57'05.5"	E 92°59'24.3"	133m	Cata
34	SR015_18	N 26°57'05.6"	E 92°59'24.4"	132m	Cata
35	SR015_18	N 26°57'05.4"	E 92°59'24.7"	132m	Cata
36	SR015_18	N 26°57'05.6"	E 92°59'25.4"	132m	Cata
37	SR015_18	N 26°57'05.8"	E 92°59'25.6"	133m	Cata
38	SR015_18	N 26°57'05.9"	E 92°59'26.2"	133m	Cata
39	SR015_18	N 26°57'06.3"	E 92°59'26.7"	133m	Cata
40	SR015_18	N 26°57'07.1"	E 92°59'27.3"	133m	Cata
41	SR015_18	N 26°57'08.2"	E 92°59'29.4"	133m	Cata
42	SR015_18	N 26°57'08.4"	E 92°59'30.0"	128m	Cata
43	SR015_18	N 26°57'14.9"	E 92°59'31.8"	122m	Cata
44	SR015_18	N 26°57'16.6"	E 92°59'32.2"	122m	Cata
45	SR015_18	N 26°57'16.2"	E 92°59'33.4"	122m	Cata
46	SR015_18	N 26°57'09.5"	E 92°59'32.5"	123m	Cata
47	SR015_18	N 26°56'25.6"	E 92°59'01.4"	117m	Cata

48	SR015_18	N 26°56'27.1"	E 92°59'02.6"	119m	Cata
49	SR015_18	N 26°56'28.1"	E 92°59'02.9"	119m	Cata
50	SR015_18	N 26°56'29.2"	E 92°59'03.6"	120m	Cata
51	SR015_18	N 26°56'30.3"	E 92°59'05.2"	120m	Cata
52	SR015_18	N 26°56'30.9"	E 92°59'04.7"	122m	Cata
53	SR015_18	N 26°56'31.8"	E 92°59'06.2"	122m	Cata
54	SR015_18	N 26°56'32.2"	E 92°59'06.6"	122m	Cata
55	SR015_18	N 26°56'32.2"	E 92°59'07.0"	122m	Cata
56	SR015_18	N 26°56'32.8"	E 92°59'07.7"	122m	Cata
57	SR015_18	N 26°56'33.5"	E 92°59'09.3"	122m	Cata
58	SR015_18	N 26°56'37.9"	E 92°59'09.2"	123m	Cata
59	SR015_18	N 26°56'38.3"	E 92°59'08.7"	123m	Cata
60	SR015_18	N 26°56'39.6"	E 92°59'11.1"	123m	Cata
61	SR015_18	N 26°56'40.2"	E 92°59'12.6"	122m	Cata
62	SR015_18	N 26°56'54.5"	E 92°59'10.2"	126m	Cata
63	SR015_18	N 26°56'56.2"	E 92°59'11.0"	126m	Cata
64	SR015_18	N 26°56'20.2"	E 92°58'57.3"	145m	Cata

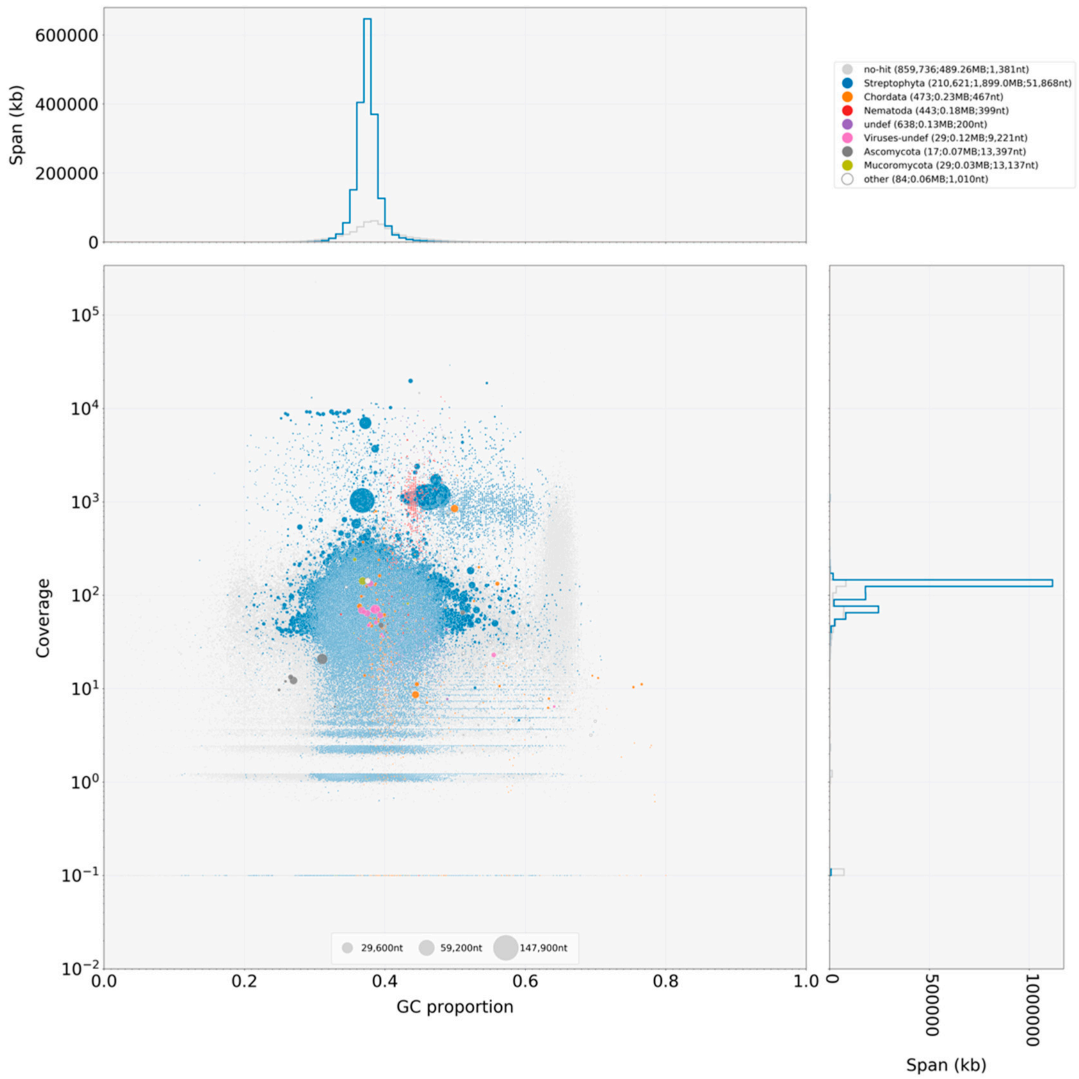

Figure S1: Blobplot for the *Alpinia nigra* reads using Blobtools v1.1.1. This plot was produced using the hits obtained from blastn and Diamond hits of NCBI nucleotide or UniProt proteome databases. These hits were used to assign taxonomy (using bestsumorder). 97.69% of the reads mapped back to the assembly out of which 84.79% belonged to Streptophyta and 12.73% were no-hits. The values in the brackets of the legend represent count, sum length and n50.

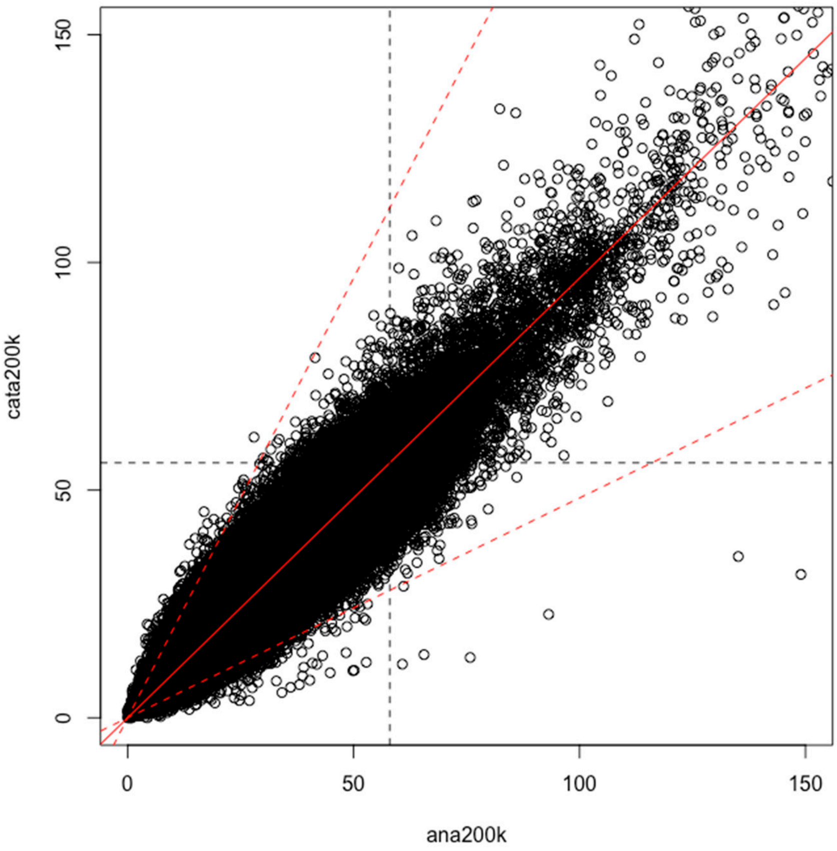

Figure S2: A comparison of the paired mapping depth per contig of the anaflexistylous and cataflexistylous pools of the 200,000 longest contigs ordered by length. The solid red line indicates line of equal depth and the dashed red lines indicate 1/2x and 2x coverage difference. The dashed black lines indicate expected mapping depths. The reference assembly is of a cataflexistylous individual. There are a few sequences with ½ coverage in ana but these might be noise due to the presence of short scaffolds.
